# Alteration of resting-state functional connectivity network properties in patients with social anxiety disorder after virtual reality-based self-training

**DOI:** 10.3389/fpsyt.2022.959696

**Published:** 2022-09-20

**Authors:** Hun Kim, Byung-Hoon Kim, Min-Kyeong Kim, Hyojung Eom, Jae-Jin Kim

**Affiliations:** ^1^Institute of Behavioral Sciences in Medicine, Yonsei University College of Medicine, Seoul, South Korea; ^2^Department of Psychiatry, Yonsei University College of Medicine, Seoul, South Korea

**Keywords:** social anxiety disorder, virtual reality, self-training, resting-state, fMRI, network analysis

## Abstract

Social anxiety disorder (SAD) is a mental disorder characterized by excessive anxiety in social situations. This study aimed to examine the alteration of resting-state functional connectivity in SAD patients related to the virtual reality-based self-training (VRS) which enables exposure to social situations in a controlled environment. Fifty-two SAD patients were randomly assigned to the experimental group who received the VRS, or the control group who did not. Self-report questionnaires and resting-state functional magnetic resonance imaging (fMRI) were performed to assess clinical symptoms and analyze the resting-state network properties, respectively. Significant decrease in social anxiety and an increase in self-esteem was found in the experimental group. From the resting-state fMRI analysis, alteration of local network properties in the left dorsolateral prefrontal gyrus (-10.0%, *p* = 0.025), left inferior frontal gyrus (-32.3%, *p* = 0.044), left insula (-17.2%, *p* = 0.046), left Heschl's gyrus (-21.2%, *p* = 0.011), bilateral inferior temporal gyrus (right: +122.6%, *p* = 0.045; left:−46.7%, *p* = 0.015), and right calcarine sulcus (+17.0%, *p* = 0.010) were found in the experimental group. Average shortest path length (+8.3%, *p* = 0.008) and network efficiency (-7.6%, *p* = 0.011) are found to be altered from the global network property analysis. In addition, the experimental group displayed more positive and more negative changes in the correlation trend of average shortest path length (*p* = 0.004) and global network efficiency (*p* = 0.014) with the severity of social anxiety, respectively. These results suggest potential effectiveness of the VRS, which is possibly related to the change of aberrant processing and control of visual and auditory linguistic stimuli and the adaptive change in rumination pattern.

## Introduction

Social anxiety disorder (SAD) is a mental disorder characterized by excessive anxiety in social situations and fear of negative evaluations from other people to an extent that these impair normal functioning in ordinary life (1). The hardships that SAD patients experience in social contexts significantly undermine their ability to plan appropriate strategies to attain their goals (2). Although SAD patients seek for social belonging, their pathological patterns of thoughts, including fear of negative evaluations from others and recurrent negative imageries of past experiences, hamper them from forming intimate relationships with other people (3, 4). Unfortunately, pathophysiology of the SAD still remains not fully understood which limits the exploration and development of effective treatment options (5).

Functional magnetic resonance image (fMRI) studies of SAD patients have been performed to explore the functional neural correlates of SAD. One of the key findings that has been consistently reported through these studies is abnormal functional connectivity between the limbic and frontal regions (6, 7). For example, alterations in resting-state functional connectivity of the amygdala with the ventromedial prefrontal cortex, dorsomedial prefrontal cortex, and orbitofrontal cortex were suggested to be associated with an increase in anxiety to social interaction, self-criticism, and difficulties of emotion regulation in SAD patients (7–10). In addition to the abnormality of limbic-frontal connectivity, aberrant functioning of brain regions associated with processing of sensory stimuli had also been suggested. Specifically, SAD patients displayed an abnormal activation level in the fusiform gyrus, the brain region involved in high-level visual cognition and face recognition (11–13). Furthermore, SAD patients showed higher activation level in the primary visual cortex when they viewed themselves from the third person's point of view, implying an excessive focus on the visual stimuli of SAD patients in social situations (14). Also, SAD patients showed an aberrant processing of auditory and linguistic stimuli in addition to visual ones. Activations of brain regions that process external speech, including the temporal gyrus and temporal pole, were significantly correlated with the extent of social anxiety when presented with human voice, while the correlations were weak or non–existent for other types of acoustic stimuli (15).

Given that the characteristics of the functional connectivity can be analyzed from the perspective of the whole network, graph theoretic analysis of network properties are being recently studied in SAD (16, 17). These literatures suggest that there exist alterations of the network metrics at both local and global scales in SAD patients, suggesting that the disrupted functional connectivity as a network can be related to the pathophysiology of the illness. More specifically, increase in the average shortest path length and the decrease in the clustering coefficient is found in the brain network of SAD, suggesting a change in interconnectivity and information transmission ability at global scale (16). Another study has reported increased nodal centralities in parahippocampal gyrus and precuneus, and decreased nodal centralities in calcarine sulcus and dorsolateral prefrontal cortex, suggesting increased level of local network connectivity within the limbic area and the decreased level of connectivity in the sensory processing area (17).

The National Institute for Health and Care Excellence (NICE) guidelines state that pharmacological treatment and psychological interventions such as the Cognitive Behavioral Therapy (CBT) are effective first-line treatments for SAD (18). However, modeling feared social stimuli and relevant social situations in clinical settings has its limitations. The use of virtual reality (VR) technology resolves the problem by reproducing relevant social situations, enabling patients to confront feared social contexts in environments with more control on situational elements (19). Given these strengths of VR, we developed a VR-based exposure therapy program that can be performed on a self-led basis in which participants can receive therapy sessions at any desired places without formally visiting clinics (20). Although our VR self-training (VRS) program provides only the exposure of feared stimuli among the full CBT sessions, exposure therapy itself is also known to be an effective treatment option of SAD (21). In addition, this form of self-training has advantages in patient accessibility and confidentiality unlike the traditional CBT which the participants physically encounter psychiatrists and therapists (22, 23).

Neuroimaging evidence further support the clinical effectiveness of repeated exposure of avoidant stimuli in SAD patients, showing normalization of functional activity and connectivity in response to the exposure treatment (24–26). More specifically, normalization of resting-state functional connectivity between the amygdala and frontal area is found after exposure-based intervention in SAD patients (10). In addition, the 2-week VR-based exposure self-training of SAD patients has demonstrated effectiveness on improving performances in social circumstances, which is found to be associated with changes in the functional activity of the visual cortices and thalamus (27). Nevertheless, few studies have explored the effect of VR-based exposure self-training on resting-state functional connectivity in SAD patients, which has been shown to be different from that of healthy controls (16, 17). In this study, we aimed to examine the change of resting-state functional connectivity related to the VR exposure self-training in SAD patients, particularly focusing on the network properties using graph theory. Participants with SAD were recruited and analyzed on their resting-state fMRI data by comparing the differences in network properties with respect to the presence or absence of the VRS. The network properties were computed in both local and global scales with the network constructed from functional connectivity, which consists of nodes as the brain regions of interest (ROIs) and edges as the connections between these ROIs (28).

Our hypothesis was that the treatment of SAD patients with the VRS could improve clinical symptoms related to the anxiety and fear in social contexts. Regarding the local network properties of specific brain regions, we expected that the amygdala and frontal regions would show lower level of network efficiency after the VRS, since these regions have shown increased level of connectivity in SAD in previous studies (29, 30). In addition, we expected that areas related to sensory processing would show increased degree centrality after the VRS based on previous evidences (16, 17). For the global network properties, we hypothesized that the small-worldness would be increased in experimental group after the VRS, reflecting altered economy of information processing in brain network.

## Materials and methods

### Participants

A total of 115 adults volunteered to participate in the study and submitted application forms *via* email after watching the internet advertisement. Participants aged between 19–30 years old were evaluated by a professional psychiatrist (M.-K. K.) based on the DSM-5 criteria for SAD on symptoms as well as demographics, and scores higher than 30 points on the Liebowitz Social Anxiety Scale (31) were used as the inclusion criteria. The exclusion criteria included: (i) history of diagnosis of major psychiatric disorders including bipolar disorder, organic mental disorder, psychotic disorder, or substance use disorder, (ii) current usage or past experience of any forms of psychiatric treatments, (iii) inability to go through MRI scanning, (iv) pregnancy, and (v) left-handedness. Sixty-one participants had met the inclusion criteria, but 9 of them were disqualified on the exclusion criteria, leaving 52 participants as eligible. The Institutional Review Board (IRB) at Gangnam Severance Hospital, Yonsei University had approved the study procedure. Written informed consents were obtained from all participants.

### Experimental process

**Figure 1:**
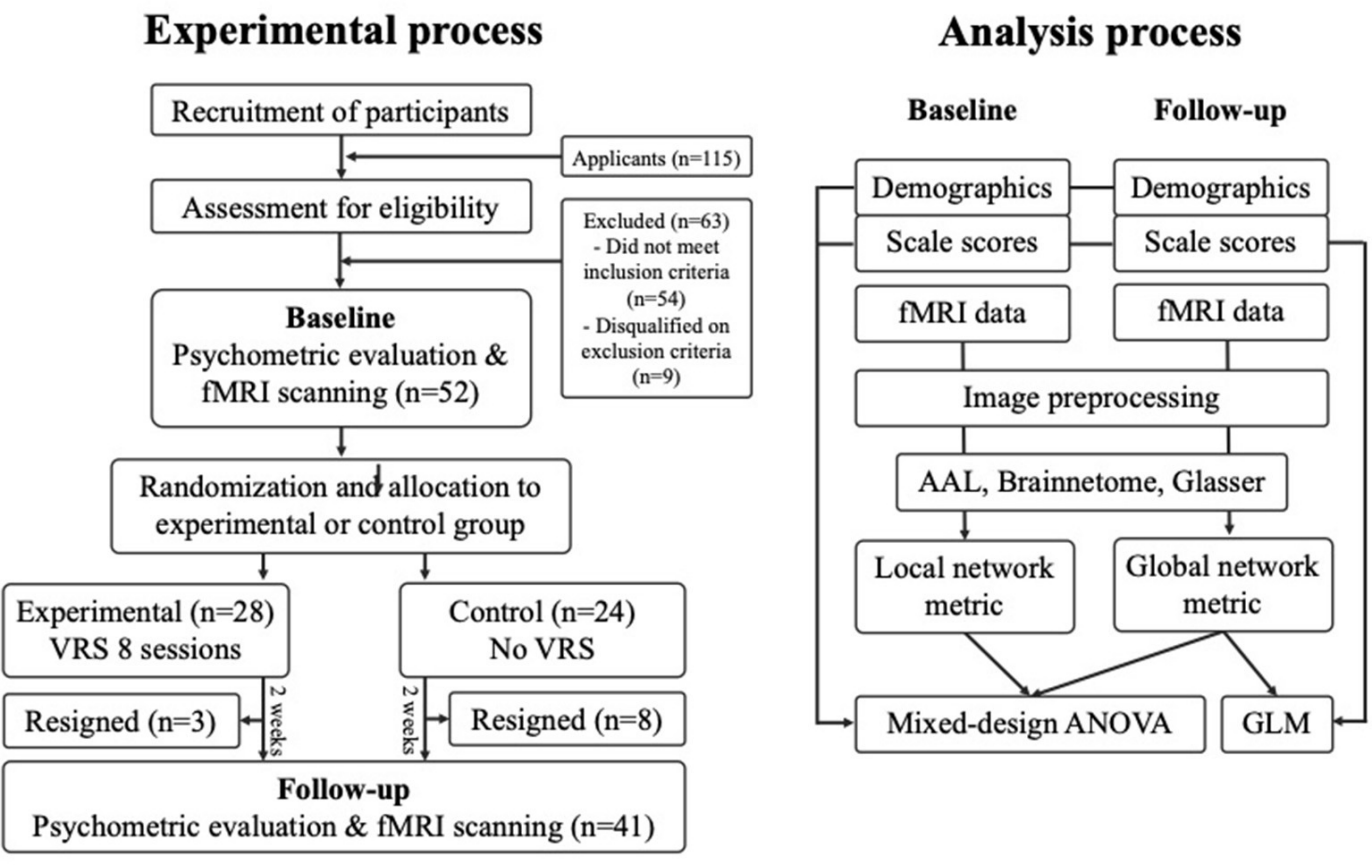
Flow chart of the experimental process.

All participants completed the initial psychometric scale evaluation using the Liebowitz Social Anxiety Scale [LSAS; (31)], Brief Fear of Negative Evaluation scale [BFNE; (32)], Social Interaction Anxiety Scale [SIAS; (33)], Rosenberg Self-Esteem Scale [RSES; (34)], and Hospital Anxiety and Depression Scale [HADS; (35)]. After the completion of psychometric scale evaluations, all participants went through a baseline fMRI scanning on the same day. Using a stratified randomization on the severity of social anxiety and gender, 24 participants were assigned to the experimental group and 28 participants to the control group. Then, the experimental group received 8 sessions of the VRS over 2 weeks, while the control group did not receive any treatments within the three-week period. At the end of the VRS for the experimental group and the waiting period for the control group, both groups completed the same psychometric questionnaires and went through a follow-up fMRI scanning. In the experimental group, three participants decided to stop receiving the VRS, leaving 21 participants who completed the whole procedure. Eight participants in the control group resigned during the waiting time, leaving 20 participants completed the follow-up fMRI scanning session. A schematic flow chart of the experimental process is depicted in Figure 1.

### VR self-training interventions

The VRS program consisted of 36 social topics total, which could be grouped into 12 situations from three environments. The three environmental contexts were daily life, school life and business life, where each context included four different situations. Each situation was further specified into three topics, where different topics have different numbers of virtual avatars and various levels of difficulties. The difficulty level increased as the number of virtual avatars became larger. The contents were displayed on a head-mounted display (HMD), consisting of a Samsung Gear VR and a Samsung Galaxy S6. With the displayer, participants went through the self-led VR training by performing speech tasks as directed by the narrations in the VR content. Samsung Gear S2 was used to measure the heart rates of participants during the training sessions. After each task, the performance of participants was evaluated based on the following criteria: the heart rate changes from the baseline to the end of the task, patterns of eyesight movement, length of speech, proportion of voiced time during the speech and self-reported evaluations about the extent of nervousness and confidence after each topic. Participants could see their scores of each measure of the performance evaluation. A sample video of the VRS contents is provided at the following link: https://youtu.be/LxfSPaSJSTE. For further details about VRS, refer to our previous study (23).

Although the contents of the VRS were designed so that the recipients could perform the training by themselves, the participants visited the VR clinic at the Gangnam Severance Hospital during the sessions to ensure that the intervention was being provided correctly. Participants were asked to complete one situation on the first session and were asked to complete two situations for the seven following sessions; these two situations for the last seven sessions included a repetition of one previously-performed situation from the preceding session and a new one, leading to a completion of eight situations and 24 topics in total.

### Image acquisition and preprocessing

Siemens Magnetom Verio 3T scanner (Siemens Medical Solutions, Erlangen, Germany) was used to collect the MRI data. Participants were guided to relax and rest for 5 min with eyes closed but not sleeping. Functional images were acquired with a gradient echo planar imaging sequence with these parameters: repetition time, 2,000 ms; echo time, 30 ms; flip angle, 90°; number of slices, 30; slice thickness, 3 mm; and matrix size, 64 × 64. Three scans were discarded before the acquisition of image to reach signal equilibrium. T1-weighted structural images were obtained with a 3D spoiled-gradient-recall sequence with the following parameters: repetition time, 1,900 ms; echo time, 2.46 ms; flip angle, 9°; number of slices, 176; slice thickness, 1 mm; and matrix size, 256 × 256. Preprocessing of the acquired images were performed using fMRIPrep v20.2.1 (36). The preprocessing of T1-weighted structural images included brain extraction, tissue segmentation, surface extraction and spatial normalization, while the preprocessing of blood-oxygen-level-dependent (BOLD) functional images included head motion correction, slice timing correction and co-registration. Further details on image data preprocessing are provided in the Supplementary material.

### Network analysis of the functional connectivity network

We examined the network properties of functional connectivity from the preprocessed fMRI data with the GRETNA toolbox (28). From the functional brain images, mean timeseries within the 90, 246, and 360 regions of the Automated Anatomical Labeling (AAL) (37), Brainnetome (38), and Glasser (39) atlases were obtained, respectively. The cerebellum and pons were excluded from the AAL (37). Functional connectivity values of every possible pair of the regions within each atlas were acquired by calculating the Pearson's correlation coefficients between the mean timeseries across time. Through these procedures, an *N* × *N* resting-state functional connectivity matrix for each participant could be defined, where *N* refers to the number of ROIs.

To compute local and global network property measures, the functional connectivity matrices were first binarized by considering the top k-percentile values as connected and others unconnected, where k?{5,10,15,20,25,30,35,40,45,50}. The binarized functional connectivity matrices served as the adjacency matrices of simple graphs, from which the network properties were estimated. The local network property metrics included the betweenness centrality, degree centrality, nodal clustering coefficient, nodal efficiency, local efficiency, and nodal shortest path. The global network metrics, which represent the characteristics of a brain network as whole, included the network efficiency, average shortest path length, and small-worldness (40). Aggregation as area under curve (AUC) with respect to the differing value of k for each network property metric was calculated. We further report network analysis results from weighted, or un-binarized functional connectivity matrices in the Supplementary material.

### Statistical analysis

Statistical analyses were performed using Python 3.8.5 with packages pandas, statsmodels and pingouin. For the demographics of the participants, an independent samples *t*-test was conducted to evaluate the difference in age between the two groups while Pearson's chi-squared test was conducted to assess the difference in gender distribution. For the psychometric scale scores of each of six self-reported questionnaires, a mixed-design analysis of variance (ANOVA) was conducted to compare the change in scores across time (baseline/follow-up) between the two groups.

The mixed-design ANOVA was also conducted to analyze the interaction effect of group-by-time on local and global network metric measures, for each individual threshold value and AUC of all threshold values. To evaluate whether a change in correlation existed between the level of social anxiety and the AUC aggregated global network metrics in response to the VRS, we constructed a general linear model on each network metric and the psychometric scale scores LSAS, BFNE, and SIAS across time of the two groups, and fitted the model with the ordinary least square solver. Here, the three scales are known to measure the level of social anxiety. For the local network metrics, we applied the false discovery rate (FDR) correction to account for the multiple comparisons problem.

## Results

### Demographics and self-assessment scales

Statistical tests did not show any significant difference of age and gender distribution between the two groups (Table 1). For the psychometric mixed-design ANOVA results of the psychometric scales, a statistically significant interaction effect between scale scores and time was found for LSAS, BFNE, RSES, and SIAS, but not for the scores of HADS-Anxiety and HADS-Depression subscale scores. A significant main effect of time was revealed for the scores of LSAS, BFNE, SIAS, HADS-Anxiety, and HADS-Depression. A *post-hoc* analysis of the paired-*t* test confirmed that there existed a statistically significant increase in RSES (t_20_ = −2.94, *p* = 0.004) and decrease in LSAS (t_20_ = 4.12, *p* < 0.001), BFNE (t_20_ = 3.92, *p* < 0.001), and SIAS (t_20_ = 5.52, *p* < 0.001) scores in the experimental group after the VRS.

**Table 1:** Results of statistical analyses on age, gender and self-report questionnaire scores of participants.

	**Control**		**Experimental**		**t /** **χ**^**2**^		***P***
**Age**	23.18 (2.04)		23.75 (2.64)		−0.86		**0.393**
**Gender (F/M)**	10 / 18		10 / 14		0.02		**0.878**
	**Baseline**	**Follow-up**	**Baseline**	**Follow-up**	**Effect**	**F**	***P***
**LSAS**	68.11 (18.94)	68.85 (20.25)	73.50 (22.24)	53.0 (23.61)	Group	1.45	0.236
					Time	11.33	0.002*
					Interaction	5.84	0.020*
**BFNE**	45.107 (7.83)	43.10 (7.39)	45.63 (5.76)	38.62 (8.46)	Group	0.36	0.552
					Time	12.15	0.001*
					Interaction	10.30	0.003*
**RSES**	16.50 (5.18)	16.50 (4.95)	14.92 (5.83)	17.86 (6.52)	Group	0.01	0.930
					Time	4.94	0.032
					Interaction	8.55	0.006*
**SIAS**	43.0 (13.28)	40.75 (13.21)	47.71 (11.06)	35.48 (15.46)	Group	0.03	0.869
					Time	29.44	<0.001*
					Interaction	11.70	0.001*
**HADS-anxiety**	8.82 (2.89)	8.25 (3.01)	10.21 (4.22)	8.29 (3.95)	Group	0.27	0.606
					Time	6.86	0.013*
					Interaction	1.01	0.322
**HADS-depression**	8.54 (3.67)	7.70 (3.54)	8.17 (4.74)	6.33 (3.85)	Group	0.77	0.385
					Time	8.13	0.007*
					Interaction	0.48	0.494

LSAS, Liebowitz Social Anxiety Scale; BFNE, Brief Fear of Negative Evaluation; RSES, Rosenberg Self Esteem Scale; SIAS, Social Interaction Anxiety Scale; HADS, Hospital Anxiety and Depression Scale. Asterisks indicate p < 0.05. Average values of the control and the experimental groups are presented with standard deviation in the round brackets.

### Local network property analysis

**Figure 2:**
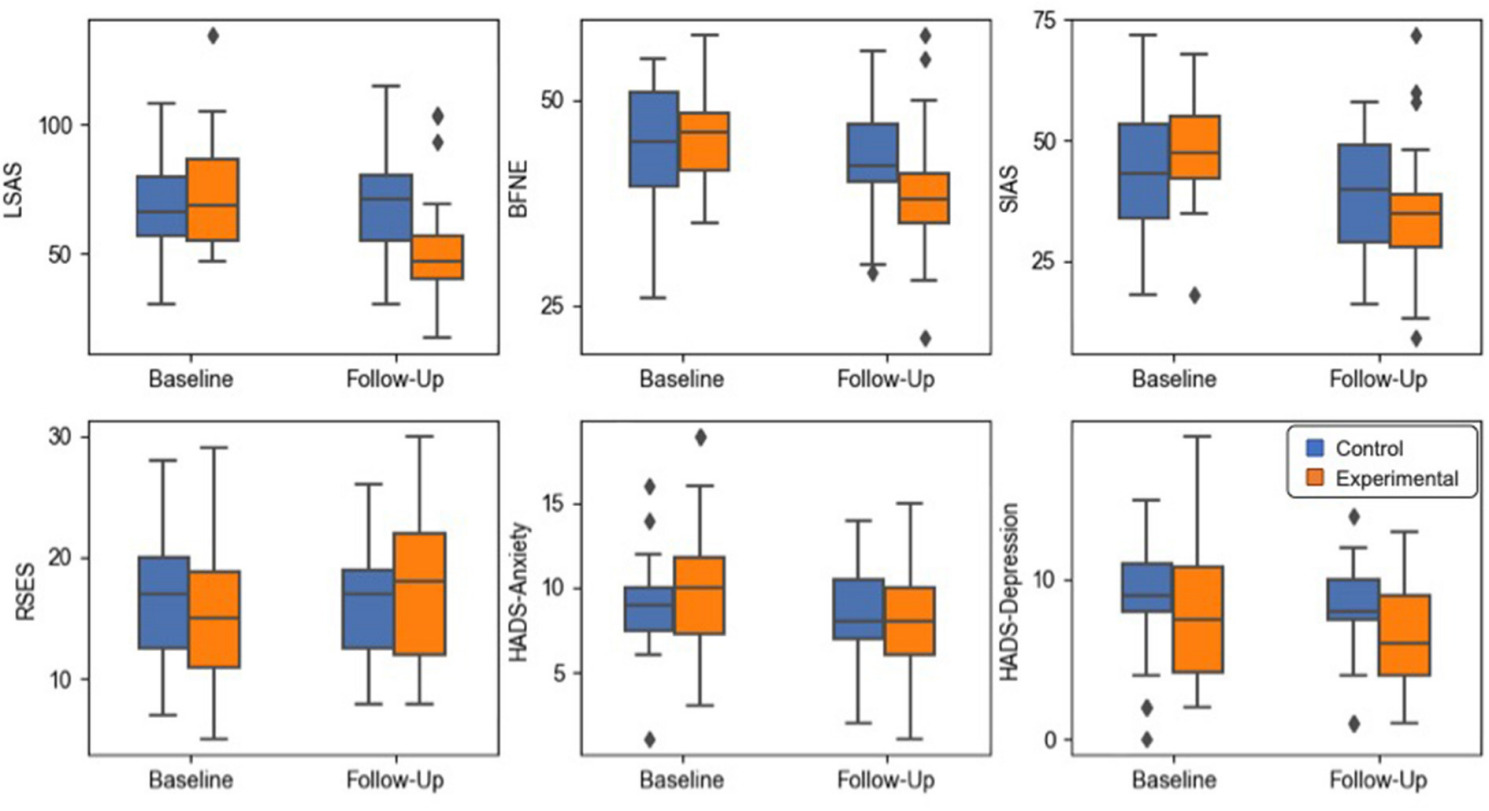
Box plots of the psychometric scale score results. The whiskers indicate the farthest data from the box within the 1.5 inter-quartile range.

Results of the local network property analysis are demonstrated in the Figure 2, Table 2.

**Table 2:** Significant results of the mixed-design ANOVA for the local network metrics at different thresholds.

**Region**	**Side**	**Network metrics**	**Threshold**	**F_1, 37_**	***p*-FDR**	**Post-hoc**	
						**t_**19**_**	***P***
**AAL**							
Inferior frontal gyrus	Left	Nodal efficiency	0.1	12.82	0.044	2.37	0.014
Heschl's gyrus	Left	Clustering coefficient	0.15	18.57	0.011	2.80	0.006
			AUC	15.22	0.035	1.54	0.070
		Local efficiency	0.15	17.80	0.014	2.66	0.008
			AUC	10.30	0.049	1.47	0.079
		Nodal efficiency	0.1	12.81	0.044	2.22	0.019
Inferior temporal gyrus	Left	Degree centrality	0.2	14.81	0.020	2.24	0.019
			0.25	15.61	0.015	2.18	0.021
			0.3	14.02	0.024	1.88	0.038
Calcarine sulcus	Right	Degree centrality	0.2	17.69	0.014	−2.51	0.011
			0.25	18.73	0.010	−2.40	0.014
			0.3	14.43	0.023	−2.14	0.023
**Brainnetome**							
BA9/46d	Left	Degree centrality	0.45	17.10	0.045	3.25	0.002
			0.5	19.01	0.025	3.36	0.002
BA20cl	Right	Degree centrality	0.45	15.41	0.045	−4.04	<0.001
			0.5	15.30	0.045	−4.07	<0.001
**Glasser**							
BA9/46d	Left	Nodal efficiency	0.15	16.37	0.027	3.69	<0.001
Middle insular area	Left	Nodal efficiency	0.15	19.81	0.046	3.53	0.001

AUC, Area under the curve; AAL, Automated Anatomical Labeling; BA9/46d, Dorsal part of the Brodmann area 9/46; BA20cl, Caudolateral part of the Brodmann area 20.

#### AAL 90: Sensory processing regions revealed

A significant interaction effect was found for the nodal efficiency of the opercular part of left inferior frontal gyrus at threshold 0.1. The left Heschl's gyrus showed significant results for the clustering coefficient at threshold 0.15 and the AUC aggregated value, the local efficiency at threshold 0.15 and the aggregated value and the nodal efficiency at threshold 0.1. The left inferior temporal gyrus and right calcarine sulcus showed significant interaction effects from the mixed-design ANOVA on degree centrality at thresholds 0.2, 0.25, and 0.3.

A *post-hoc* paired *t*-test within the experimental group indicated a significant decrement of the nodal efficiency on the opercular part of left inferior frontal gyrus. For the left Heschl's gyrus, a significant decrement of the clustering coefficient, local efficiency and the nodal efficiency was found at thresholds 0.15, 0.15, and 0.1. Significant decrement and increment of the degree centrality were found in the left inferior temporal gyrus and right calcarine sulcus, respectively.

#### Brainnetome 246: Dorsolateral prefrontal cortex revealed

Significant interaction effects were found for the degree centrality of the left dorsal part of the Brodmann area 9/46 (BA9/46d) at threshold 0.45, 0.5 and the right caudolateral part of the Brodmann area 20 (BA20cl) at threshold 0.45, 0.5 which lies within the left dorsolateral prefrontal cortex and the right inferior temporal gyrus, respectively.

The *post-hoc* paired *t*-test demonstrated significant decrement and increment of degree centrality at threshold 0.45 in the BA9/46d (t = 3.25, *p* = 0.002) and the BA20cl (t = −4.04, *p* < 0.001) for the experimental group, respectively. For degree centrality at threshold 0.50, the *post-hoc* paired *t*-test demonstrated significant decrement and increment in the BA9/46d (t = 3.36, *p* = 0.002) and the BA20cl (t = −4.07, *p* < 0.001) for the experimental group, respectively.

#### Glasser 360: Middle insular area revealed

Significant interaction effects were found for the nodal efficiency of the left BA9/46d and the left middle insular area at threshold 0.15. The *post-hoc* paired *t*-test indicated significant decrement of the nodal efficiency at both regions (left BA9/46d, t = 3.69, *p* < 0.001; left middle insular area, t = 3.53, *p* = 0.001) in the experimental group.

### Global network property analysis

**Figure 3:**
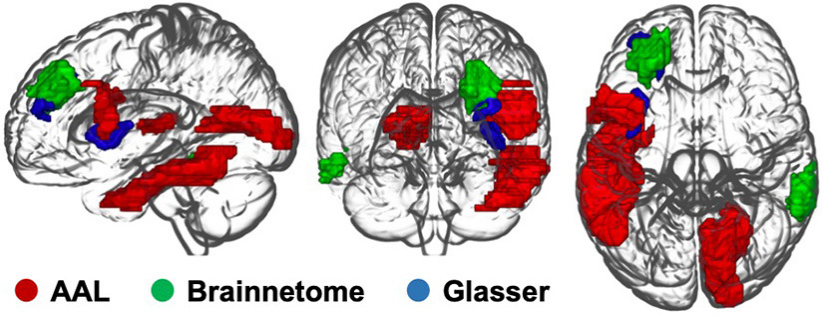
Brain regions with significant interaction effects of the local network metrics.

**Figure 4:**
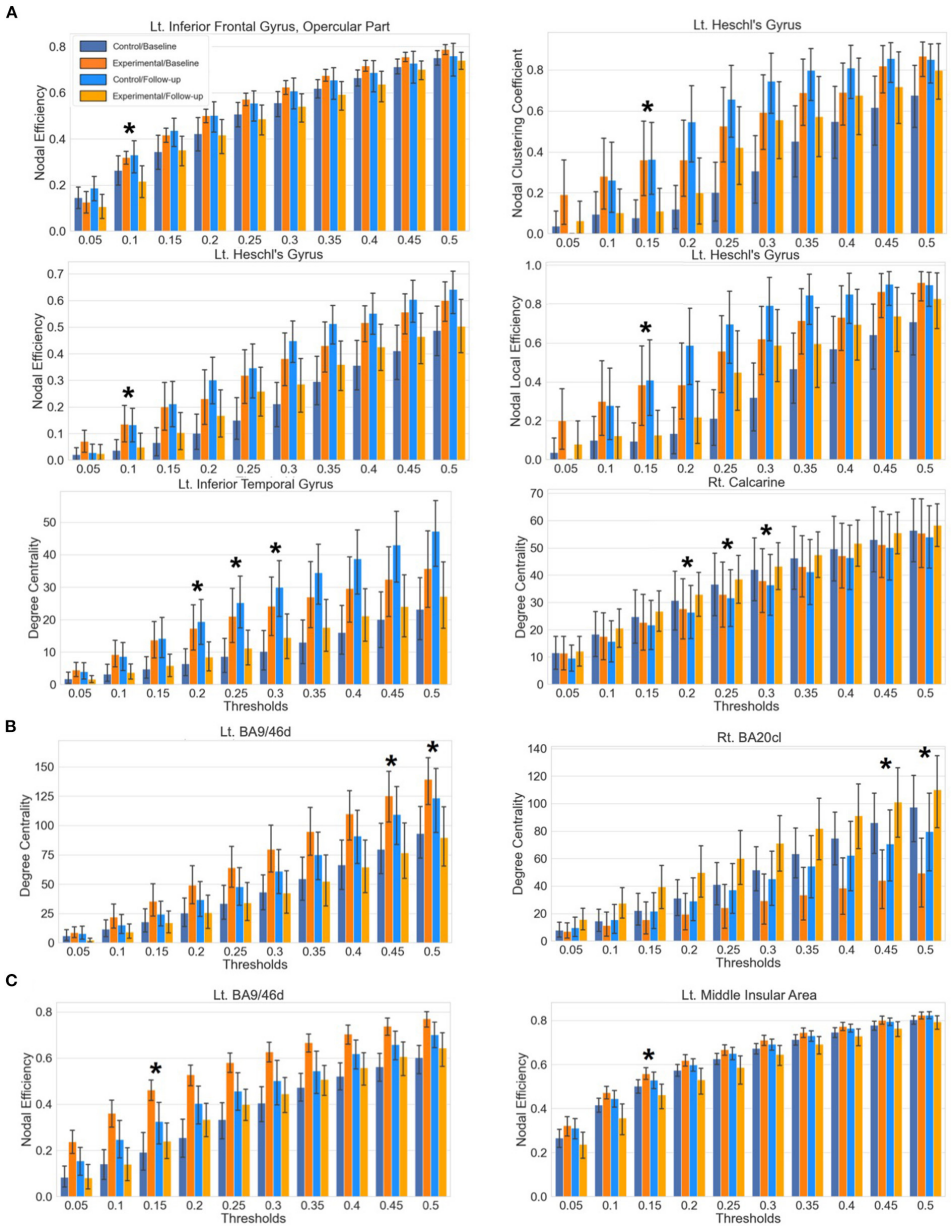
Bar plots of the local network metrics at baseline and follow-up with respect to varying thresholds. Statistically significant results after the correction of multiple comparisons are indicated with an asterisk. Each whisker indicates the 95% confidence interval. **(A)** Results from the AAL atlas. **(B)** Results from the Brainnetome atlas. **(C)** Results from the Glasser atlas.

Results of the global network property analysis are demonstrated in the Figures 3, 4 and Table 3.

**Table 3:** Significant results of the mixed-design ANOVA for the global network metrics at different thresholds.

**Network metrics**	**Threshold**	**F_1, 37_**	***p***	**Post-hoc**	
				**t_**19**_**	***p***
**Brainnetome**					
Network efficiency	AUC	4.53	0.040	1.29	0.106
	0.05	7.21	0.011	1.72	0.051
	0.1	6.42	0.016	1.69	0.054
	0.15	6.06	0.019	1.64	0.059
	0.2	5.00	0.031	1.40	0.090
Average shortest path length	AUC	7.36	0.010	−1.61	0.062
	0.05	6.73	0.014	−1.66	0.057
	0.1	7.89	0.008	−1.73	0.050
	0.15	7.64	0.009	−1.68	0.055
	0.2	5.75	0.022	−1.39	0.090
	0.25	4.54	0.040	−1.16	0.131
**Glasser**					
Network efficiency	0.05	4.42	0.042	1.00	0.166
Average shortest path length	AUC	4.31	0.045	−1.15	0.131

#### AAL 90

**Figure 5:**
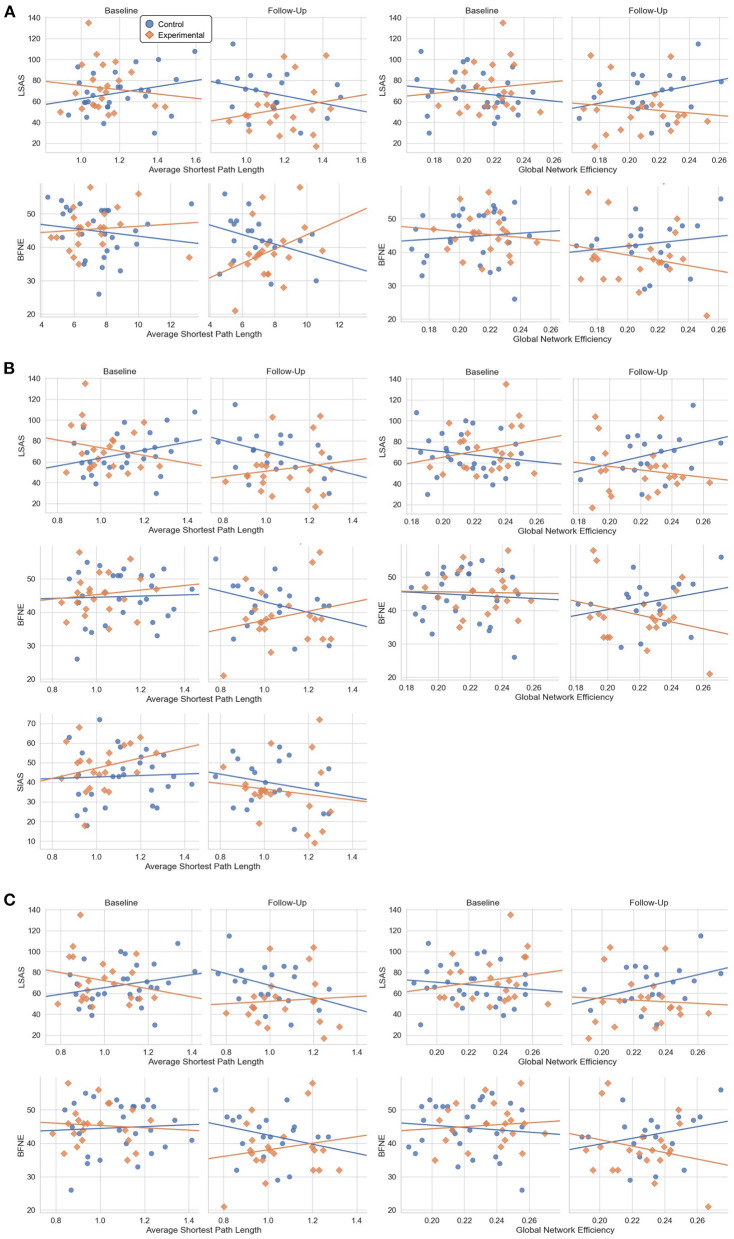
Scatter plot between the psychometric scale scores and the global network metrics at baseline and follow-up which showed significant results from the general linear model analysis. **(A)** Results from the AAL atlas. **(B)** Results from the Brainnetome atlas. **(C)** Results from the Glasser atlas.

Mixed-design ANOVA with the global network property metrics did not show any significant results. The general linear model analysis revealed that the experimental group displayed more positive correlation between the average shortest path length and the LSAS score (t = 2.39, *p* = 0.019) and more negative correlation between the global network efficiency and the LSAS score (t = −2.18, *p* = 0.032), in response to the VRS Figure 5. A similar result was found also for the BFNE score, indicating a more negative correlation with the global network efficiency (t = 2.20, *p* = 0.031) after the VRS in the experimental group. *Post-hoc z*-test between the correlation coefficient at baseline and at follow-up within the experimental group did not show significant results.

#### Brainnetome 246

A significant interaction effect was found for the global network efficiency at threshold 0.05 and the average path length at AUC aggregated threshold from the mixed ANOVA. *Post-hoc* paired *t*-test within the experimental group suggested insignificant change between the baseline and the follow-up for both network metrics.

From the general linear model, a more positive correlation between the average shortest path length and the LSAS (t = 2.78, *p* = 0.007), BFNE (t = 2.706, *p* = 0.008), and SIAS (t = 2.21. *p* = 0.03) and more negative correlation between the global network efficiency and the LSAS (t = −2.52. *p* = 0.014) and BFNE (t = −2.55. *p* = 0.013) were found. *Post-hoc z*-test of the correlation coefficient did not show significant change in in the experimental group.

#### Glasser 360

From the mixed-design ANOVA, significant interaction effects were found for the average shortest path length at thresholds 0.05, 0.1, 0.15, 0.2, 0.25, and the AUC aggregated value and the network efficiency at thresholds 0.05, 0.1, 0.15, 0.2 and the AUC aggregated value. *Post-hoc* paired *t*-test did not indicate any significant change of the average shortest path length and network efficiency before and after the intervention in the experimental group.

The change in correlation followed a similar trend to the results of the Brainnetome atlas, indicating a more positive correlation between the average shortest path length and the LSAS (t = 2.99, *p* = 0.004) and BFNE (t = 2.91, *p* = 0.005), and more negative correlation between the global network efficiency and the LSAS (t = −2.48. *p* = 0.015) and BFNE (t = −2.57. *p* = 0.012). No significant result was found for the *post-hoc z*-test of the correlation coefficient change in the experimental group.

## Discussion

In this study, we aimed to examine the effect of the VRS, which only presents VR-based exposure treatment sessions, in relieving clinical symptoms of SAD and investigate related changes of resting-state functional connectivity in terms of global and local network properties. A significant decrease in LSAS, BFNE, and SIAS scores and an increase in RSES score were observed in SAD patients who went through the VRS compared to those in the control group. The network analyses of resting-state functional connectivity with graph theoretical approach showed decrease of the nodal efficiency on the opercular part of left inferior frontal gyrus, decrease of the clustering coefficient, local efficiency and the nodal efficiency on the left Heschl's gyrus, decrease of the degree centrality on the left inferior temporal gyrus, and increase of the degree centrality on the right calcarine sulcus after the VRS. From the global network property analyses, changes in the correlation trend were present between the LSAS score and the global network efficiency and between the LSAS score and the average shortest path length.

The change in self-report questionnaire scores in the experimental group indicates an improvement in subjective symptoms after the VR-based intervention. Specifically, participants who experienced the VRS significantly improved in self-esteem, anxiety in social contexts and fear of negative evaluations, compared to those who did not. These observations are consistent with previous studies, which reported a significant decrease in anxiety of social environment and internalized shame, a feeling strongly associated with perception of negative evaluations by other people, after treatment sessions with the exposure-based treatment with VR settings (41, 42). These promising results signify the clinical potential of the VRS, recently uprising as a viable alternative to the conventional exposure therapy, in the treatment of mental disorders.

From the network property analyses of resting-state functional connectivity, significant group-by-time interaction effects of local network metrics were observed in the opercular part of left inferior frontal gyrus, left Heschl's gyrus, left inferior temporal gyrus, and right calcarine sulcus for the AAL atlas. The experimental group displayed significant decrease of nodal efficiency on the opercular part of left inferior frontal gyrus, the region of language processing circuits (43, 44). Thus, the decrement of nodal efficiency, which represents the extent of communication with other brain regions, suggests that the alleviation of SAD symptoms by VRS may be associated with lower level of abnormal concentration on language comprehension of SAD patients. Significant finding from previous study, which indicates an abnormal increase in activation in SAD patients in the opercular part of left inferior frontal gyrus in response to socially oriented language cues, also supports our finding (45). The decrease in clustering coefficient, nodal efficiency, and local efficiency in the left Heschl's gyrus in the experimental group after the VRS further supports the effect of VRS in the change of language processing in social context. The decrease in these local network metrics indicates a lower level of functional connections of the left Heschl's gyrus with neighboring regions (40). The left Heschl's gyrus is involved in perception of acoustic stimulus (46) and is associated with the auditory language comprehension (47), a critical part of social interaction. Thus, an increased magnitude of functional communication of the left Heschl's gyrus and opercular part of the left inferior frontal gyrus with other brain regions may reflect the problematic processing of acoustic language comprehension with social context, which could be relieved by the VRS.

The experimental group also displayed a decline in the left inferior temporal gyrus and a rise of the degree centrality in the right calcarine sulcus after the VRS. Considering that the degree centrality reflects the centeredness in communications of a brain region with other brain, the significant changes of the degree centrality in response to the VRS suggest the abnormal mode of interaction of the right calcarine sulcus and left inferior temporal gyrus in SAD patients. Both regions constitute the ventral visual pathway (48, 49), which receives visual information from the primary visual cortex and diverges into the inferior temporal cortex. It enables the visual processing of recognition of physical shapes and objects (50, 51). Visual processing of SAD patients is known to be altered with distorted perception and understanding of visual information in social contexts, and this misunderstanding is thought to be contributing to their biased interpretation and subsequent excessive social anxiety (52, 53). The degree centrality changes in the inferior temporal gyrus and calcarine sulcus in response to the VRS may be associated with the change of the visual processing after the intervention. Furthermore, the right inferior temporal gyrus within the region BA20cl also showed a consistent result of increased degree centrality after the VRS from the Brainnetome atlas analysis.

Interestingly, significant group-by-time interaction effect of local network property was found within the left Brodmann area 9/46 for both Brainnetome and Glasser atlases. These regions constitute part of the dorsolateral prefrontal cortex, which takes central role in cognitive control. More specifically, the dorsolateral prefrontal cortex is known to communicate with the occipital area to modulate top-down sensory inhibition mechanism for anxiety in social situation, showing exaggerated network connectivity metrics in the SAD (17). Decrement of the degree centrality and the network efficiency in the BA9/46d thus can suggest that the exaggerated activity of the top-down sensory regulation becomes relieved after the VRS. Considering that the insula also takes part in the mediation and awareness of the sensory stimuli and that its activity is elevated in SAD (54), decreased nodal efficiency within the middle insular area after VRS treatment may further support the interpretation of relief in top-down sensory regulation in the SAD. The different regions revealed from the local network analysis between the AAL atlas and the Brainnetome and Glasser atlases may be due to the fact that the latter two atlases focus more on parcellating functional regions rather than anatomical ones (37–39).

The mixed-design ANOVA of the global network metrics did not show any significant interaction effect for the AAL atlas. Significant group-by-time interaction effect was found for the Brainnetome atlas and the Glasser atlas, indicating increased average shortest path length and decreased global network efficiency in the experimental group after VRS when compared to the control group. Given that the average shortest path length becomes shorter when there are nodes in the network that acts as the central hubs in the network with high centrality measures (40), the elevation of average shortest path length can reflect the fact that the degree centrality of multiple regions is found to be decreased in response to the VRS from the local network property analysis. The decrement of global network efficiency is probably related to the fact that it is mathematically defined to be inversely proportional to the average shortest path length (40). However, these interpretations may require further verification in the future since the *post-hoc* statistical analysis did not show significant results when the tests are performed within the experimental group only.

The network property analyses of brain functional connectivity of SAD patients at global scale with the general linear model indicated that the correlation trend between the LSAS score and the global network efficiency, measuring the efficiency of information transmission among different brain regions, showed a change to a more negative correlation in the experimental group at follow-up than in baseline when compared to the control group. Additionally, the correlation trend between the LSAS and the average shortest path length, representing the shortness of minimum length of path between any two nodes, implied a stronger positive statistical correlation in the experimental group at follow-up than in baseline when compared to the control group as well. This result was consistent across three different atlases and the same trend was found with the BFNE score, except for the average shortest path length within the AAL atlas. However, the *post-hoc* test did not suggest a significant difference in the correlation coefficient within the experimental group before and after the VRS.

We cautiously interpret that the change in the pattern of rumination might reflect this alteration of global network property in the experimental group due to the VRS. Rumination can be divided into the abstract rumination and the concrete rumination, based on the mode of processing (55). The former is a type of cognitive process that places more emphasis on relatively abstract and insoluble aspects of confronted problems, including retrieved memories about past experiences and the reason of the problem being faced, while the latter places more emphasis on the information specifically relevant to the context and considers more about the feasible solutions of the problem being faced. In other words, the attitude accompanied by the abstract rumination can result in exacerbating the anxiety in social situation, while the concrete rumination can oppositely help relieve social anxiety for SAD patients (55–59). The process of concrete rumination requires concurrent activation of multiple brain circuits, a process that occurs globally in the brain, and increased communication efficiency and interconnectivity between different brain regions facilitate these global-scale interactions (60). Thus, the change in the correlation trend between the global network metrics and the social anxiety scale scores in the experimental group may suggest that the VRS might have had an impact on the shift from abstract rumination to concrete rumination. Individuals with higher communication efficiency and interconnectivity of brain regions benefitted more from concrete rumination and adoption of adaptive attitudes in coping with social problems, effectively decreasing levels of social anxiety.

In contrast to our hypotheses, we were not able to observe any specific change in the local network metrics of limbic areas such as the amygdala for SAD patients who received the VRS. This discrepancy may reflect the fact that different subregions of the amygdala, including the superficial, basolateral, and ventromedial subregions, exhibit different pattern of functional connections in SAD patients (7, 60, 61). The differences in functional connectivity patterns of distinct amygdala subregions in SAD patients could have lessened the coherence of measured functional connectivity of the amygdala as a whole, leading to an insignificant statistical result in the analyses. Accordingly, further studies on resting-state functional connectivity specifically focusing on the local network properties of the subregions of the amygdala may be an interesting research topic in the future.

The strength of our study is that it is the first to analyze the effect of the VRS for SAD patients in terms of the resting-state functional connectivity network properties of the brain. Nevertheless, some limitations should be noted for this study. First, there existed a limitation on the study design. Specifically, we did not include any healthy control groups or assign any participants to the sham treatment group, which could have provided a broader interpretation for the effect of the VRS if included. Second, we did not control for the history of drug intake for the participants.

## Conclusion

We showed that intervention with the VRS alleviated the degree of social anxiety and increased self-esteem in SAD patients. From the resting-state functional connectivity analysis, change in the local network properties of the left dorsolateral prefrontal cortex, opercular part of left inferior frontal gyrus, left middle insular area, left Heschl's gyrus, bilateral inferior temporal gyrus, and right calcarine sulcus were revealed in response to the treatment. These changes may be related to the change in aberrant processing of sensory information including the auditory linguistic and visual stimuli. Altered correlation trend between the global network properties and the level of social anxiety after the VRS further suggest changes of the brain network as a whole which may reflect adaptation in the pattern of rumination.

## Data availability statement

The datasets presented in this article are not readily available because restrictions on privacy concerns. Requests to access the datasets should be directed to the corresponding author.

## Ethics statement

The studies involving human participants were reviewed and approved by the Institutional Review Board (IRB) at Gangnam Severance Hospital, Yonsei University. The patients/participants provided their written informed consent to participate in this study.

## Author contributions

Conceptualization: HK, B-HK, and J-JK. Participant evaluation and data acquisition: M-KK and HE. Formal analysis: HK and B-HK. Writing—original draft preparation: HK. Writing—review and editing and supervision: B-HK and J-JK. All authors contributed to the article and approved the submitted version.

## Funding

This work was supported by the National Research Foundation of Korea (NRF) grant funded by the Korea Government (MSIT) (No. NRF-2021M3E5D9025019).

## Conflict of interest

The authors declare that the research was conducted in the absence of any commercial or financial relationships that could be construed as a potential conflict of interest.

## Publisher's note

All claims expressed in this article are solely those of the authors and do not necessarily represent those of their affiliated organizations, or those of the publisher, the editors and the reviewers. Any product that may be evaluated in this article, or claim that may be made by its manufacturer, is not guaranteed or endorsed by the publisher.

## References

[1] American Psychiatric Association. Diagnostic and Statistical Manual of Mental Disorders (DSM-5®). Washington, DC: American Psychiatric Publishing, American Psychiatric Association. (2013).

[2] Hiemisch A Ehlers A Westermann R. Mindsets in social anxiety: a new look at selective information processing. J Behav Ther Exp Paychiatry. (2002) 33:103–14. 10.1016/S0005-7916(02)00022-8 12472174

[3] Guarnera M Buccheri SL Castellano S Di Corrado D Di Nuovo S. Social anxiety and mental imagery processes. Clin Neuropsychiatry. (2019) 16:173–81. Available online at: https://www.clinicalneuropsychiatry.org/clinical-neuropsychiatry-volume-16-issue-4-august-2019/# 34908953 PMC8650197

[4] Kim S Liu M Qiao A Miller LC. “I want to be alone, but I don't want to be lonely”: uncertainty management regarding social situations among college students with social anxiety disorder. Health Commun. (2021) 17:1–11. 10.1080/10410236.2021.1912890 33866871

[5] Fox AS Kalin NH. translational neuroscience approach to understanding the development of social anxiety disorder and its pathophysiology. Am J Psychiatry. (2014) 171:1162–73. 10.1176/appi.ajp.2014.14040449 25157566 PMC4342310

[6] Lai CH. Task MRI-based functional brain network of anxiety. Adv Exp Med Biol. (2020) 1191:3–20. 10.1007/978-981-32-9705-0_1 32002919

[7] Mizzi S Pedersen M Lorenzetti V Heinrichs M Labuschagne I. Resting-state neuroimaging in social anxiety disorder: a systematic review. Mol Psychiatry. (2022) 27:164–79. 10.1038/s41380-021-01154-6 34035474

[8] Dodhia S Hosanagar A Fitzgerald DA Labuschagne I Wood AG Nathan PJ. Modulation of resting-state amygdala-frontal functional connectivity by oxytocin in generalized social anxiety disorder. Neuropsychopharmacology. (2014) 39:2061–9. 10.1038/npp.2014.53 24594871 PMC4104324

[9] Liao W Qiu C Gentili C Walter M Pan Z Ding J. Altered effective connectivity network of the amygdala in social anxiety disorder: a resting-state FMRI study. PLoS ONE. (2010) 5:e15238. 10.1371/journal.pone.0015238 21203551 PMC3008679

[10] Yuan M Zhu H Qiu C Meng Y Zhang Y Shang J. Group cognitive behavioral therapy modulates the resting-state functional connectivity of amygdala-related network in patients with generalized social anxiety disorder. BMC Psychiatry. (2016) 16:198. 10.1186/s12888-016-0904-8 27296506 PMC4906710

[11] Ergül C Ulasoglu-Yildiz C Kurt E Koyuncu A Kicik A Demiralp T. Intrinsic functional connectivity in social anxiety disorder with and without comorbid attention deficit hyperactivity disorder. Brain Res. (2019) 1722:146364. 10.1016/j.brainres.2019.146364 31400309

[12] Weiner KS Zilles K. The anatomical and functional specialization of the fusiform gyrus. Neuropsychologia. (2016) 83:48–62. 10.1016/j.neuropsychologia.2015.06.033 26119921 PMC4714959

[13] Yu X Ruan Y Zhang Y Wang J Liu Y Zhang J. Cognitive neural mechanism of social anxiety disorder: a meta-analysis based on fMRI studies. Int J Environ Res Public Health. (2021) 18:5556. 10.3390/ijerph18115556 34067468 PMC8196988

[14] Pujol J Giménez M Ortiz H Soriano-Mas C López-Solà M Farré M. Neural response to the observable self in social anxiety disorder. Psychol Med. (2013) 43:721–31. 10.1017/S0033291712001857 22895096

[15] Kreifelts B Eckstein KN Ethofer T Wiegand A Wächter S Brück C. Tuned to voices and faces: cerebral responses linked to social anxiety. Neuroimage. (2019) 197:450–6. 10.1016/j.neuroimage.2019.05.018 31075391

[16] Zhu H Qiu C Meng Y Yuan M Zhang Y Ren Z. Altered topological properties of brain networks in social anxiety disorder: a resting-state functional MRI study. Sci Rep. (2017) 7:43089. 10.1038/srep43089 28266518 PMC5339829

[17] Yang X Liu J Meng Y Xia M Cui Z Wu X. Network analysis reveals disrupted functional brain circuitry in drug-naive social anxiety disorder. Neuroimage. (2019) 190:213–23. 10.1016/j.neuroimage.2017.12.011 29223742

[18] Pilling S Mayo-Wilson E Mavranezouli I Kew K Taylor C Clark DM. Recognition, assessment and treatment of social anxiety disorder: summary of NICE guidance. BMJ. (2013) 346:f2541. 10.1136/bmj.f2541 23697669

[19] Mishkind MC Norr AM Katz AC Reger GM. Review of virtual reality treatment in psychiatry: evidence versus current diffusion and use. Curr Psychiatry Rep. (2017) 19:80. 10.1007/s11920-017-0836-0 28920179

[20] Kim HE Hong YJ Kim MK Jung YH Kyeong S Kim JJ. Effectiveness of self-training using the mobile-based virtual reality program in patients with social anxiety disorder. Comp Human Behav. (2017) 73:614–9. 10.1016/j.chb.2017.04.017

[21] Craske MG Treanor M Conway CC Zbozinek T Vervliet B. Maximizing exposure therapy: an inhibitory learning approach. Behav Res Therapy. (2014) 58:10–23. 10.1016/j.brat.2014.04.006 24864005 PMC4114726

[22] Mitrousia V Giotakos O. Virtual reality therapy in anxiety disorders. Psychiatriki. (2016) 27:276–86. 10.22365/jpsych.2016.274.276 28114091

[23] Oing T Prescott J. Implementations of virtual reality for anxiety-related disorders: systematic review. JMIR Serious Games. (2018) 6:e10965. 10.2196/10965 30404770 PMC6249506

[24] Månsson KN Frick A Boraxbekk CJ Marquand AF Williams SC Carlbring P. Predicting long-term outcome of Internet-delivered cognitive behavior therapy for social anxiety disorder using fMRI and support vector machine learning. Transl Psychiatry. (2015) 5:e530. 10.1038/tp.2015.22 25781229 PMC4354352

[25] Månsson KN Salami A Frick A Carlbring P Andersson G Furmark T. Neuroplasticity in response to cognitive behavior therapy for social anxiety disorder. Transl Psychiatry. (2016) 6:e727. 10.1038/tp.2015.218 26836415 PMC4872422

[26] Yuan M Meng Y Zhang Y Nie X Ren Z Zhu H. Cerebellar neural circuits involving executive control network predict response to group cognitive behavior therapy in social anxiety disorder. Cerebellum. (2017) 16:673–82. 10.1007/s12311-017-0845-x 28155138

[27] Kim MK Eom H Kwon JH Kyeong S Kim JJ. Neural effects of a short-term virtual reality self-training program to reduce social anxiety. Psychol Med. (2020) 52:1296–305. 10.1017/S0033291720003098 32880252

[28] Wang J Wang X Xia M Liao X Evans A He Y. a graph theoretical network analysis toolbox for imaging connectomics. Front Human Neurosci. (2015) 9:386. 10.3389/fnhum.2015.00386 26175682 PMC4485071

[29] Brühl AB Delsignore A Komossa K Weidt S. Neuroimaging in social anxiety disorder—a meta-analytic review resulting in a new neurofunctional model. Neurosci Biobehav Rev. (2014) 47:260–80. 10.1016/j.neubiorev.2014.08.003 25124509

[30] Etkin A Wager TD. Functional neuroimaging of anxiety: a meta-analysis of emotional processing in PTSD, social anxiety disorder, and specific phobia. Am J Psychiatry. (2007) 164:1476–88. 10.1176/appi.ajp.2007.07030504 17898336 PMC3318959

[31] Fresco DM Coles ME Heimberg RG Liebowitz MR Hami S Stein MB. The liebowitz social anxiety scale: a comparison of the psychometric properties of self-report and clinician-administered formats. Psychol Med. (2001) 31:1025–35. 10.1017/S0033291701004056 11513370

[32] Leary MR. A brief version of the fear of negative evaluation scale. Pers Soc Psychol Bull. (1983) 9:371–5. 10.1177/0146167283093007

[33] Mattick RP Clarke JC. Development and validation of measures of social phobia scrutiny fear and social interaction anxiety. Behav Res Therapy. (1998) 36:455–70. 10.1016/S0005-7967(97)10031-6 9670605

[34] Rosenberg M. Society and the Adolescent Self-Image. Princeton, NJ: Princeton University Press. (1956).

[35] Zigmond AS Snaith RP. The hospital anxiety and depression scale. Acta Psychiatr Scand. (1983) 67:361–70. 10.1111/j.1600-0447.1983.tb09716.x 6880820

[36] Esteban O Markiewicz CJ Blair RW Moodie CA Isik AI Erramuzpe A. fMRIPrep: a robust preprocessing pipeline for functional MRI. Nat Methods. (2019) 16:111–6. 10.1038/s41592-018-0235-4 30532080 PMC6319393

[37] Tzourio-Mazoyer N Landeau B Papathanassiou D Crivello F Etard O Delcroix N. Automated anatomical labeling of activations in SPM using a macroscopic anatomical parcellation of the MNI MRI single-subject brain. Neuroimage. (2002) 15:273–89. 10.1006/nimg.2001.0978 11771995

[38] Fan L Li H Zhuo J Zhang Y Wang J Chen L. The human brainnetome atlas: a new brain atlas based on connectional architecture. Cereb Cortex. (2016) 26:3508–26. 10.1093/cercor/bhw157 27230218 PMC4961028

[39] Glasser MF Coalson TS Robinson EC Hacker CD Harwell J Yacoub E. A multi-modal parcellation of human cerebral cortex. Nature. (2016) 536:171–8. 10.1038/nature18933 27437579 PMC4990127

[40] Wang J Zuo X He Y. Graph-based network analysis of resting-state functional MRI. Front Sys Neurosci. (2010) 4:16. 10.3389/fnsys.2010.00016 20589099 PMC2893007

[41] Horigome T Kurokawa S Sawada K Kudo S Shiga K Mimura M. Virtual reality exposure therapy for social anxiety disorder: a systematic review and meta-analysis. Psychol Med. (2020) 50:2487–97. 10.1017/S0033291720003785 33070784

[42] Kim HJ Lee S Jung D Hur JW Lee HJ Lee S. Effectiveness of a participatory and interactive virtual reality intervention in patients with social anxiety disorder: longitudinal questionnaire study. J Med Int Res. (2020) 22:e23024. 10.2196/23024 33021481 PMC7576535

[43] Feng G Chen Q Zhu Z Wang S. Separate brain circuits support integrative and semantic priming in the human language system. Cerebral Cortex. (2016) 26:3169–82. 10.1093/cercor/bhv148 26209843

[44] Martin RC. Language processing: functional organization and neuroanatomical basis. Annu Rev Psychol. (2003) 54:55–89. 10.1146/annurev.psych.54.101601.145201 12359917

[45] Boehme S Ritter V Tefikow S Stangier U Strauss B Miltner WH. Neural correlates of emotional interference in social anxiety disorder. PLoS ONE. (2015) 10:e0128608. 10.1371/journal.pone.0128608 26042738 PMC4456154

[46] Warrier C Wong P Penhune V Zatorre R Parrish T Abrams D. Relating structure to function: Heschl's gyrus and acoustic processing. J Neurosci. (2009) 29:61–9. 10.1523/JNEUROSCI.3489-08.2009 19129385 PMC3341414

[47] Shinn AK Baker JT Cohen BM Ongür D. Functional connectivity of left Heschl's gyrus in vulnerability to auditory hallucinations in schizophrenia. Schizophrenia Res. (2013) 143:260–8. 10.1016/j.schres.2012.11.037 23287311 PMC3601525

[48] Lin JL Silva-Pereyra J Chou CC Lin FH. The sequence of cortical activity inferred by response latency variability in the human ventral pathway of face processing. Sci Rep. (2018) 8:5836. 10.1038/s41598-018-23942-x 29643441 PMC5895585

[49] Pietrini P Furey ML Ricciardi E Gobbini MI Wu WH Cohen L. Beyond sensory images: object-based representation in the human ventral pathway. Proc Natl Acad Sci USA. (2004) 101:5658–63. 10.1073/pnas.0400707101 15064396 PMC397466

[50] Baddeley R Abbott LF Booth MC Sengpiel F Freeman T Wakeman EA. Responses of neurons in primary and inferior temporal visual cortices to natural scenes. Proc Biol Sci. (1997) 264:1775–83. 10.1098/rspb.1997.0246 9447735 PMC1688734

[51] Goodale MA Milner AD. Separate visual pathways for perception and action. Trends Neurosci. (1992) 15:20–5. 10.1016/0166-2236(92)90344-8 1374953

[52] Gentili C Gobbini MI Ricciardi E Vanello N Pietrini P Haxby JV. Differential modulation of neural activity throughout the distributed neural system for face perception in patients with Social Phobia and healthy subjects. Brain Res Bulletin. (2008) 77:286–92. 10.1016/j.brainresbull.2008.08.003 18771714

[53] Liao W Chen H Feng Y Mantini D Gentili C Pan Z. Selective aberrant functional connectivity of resting state networks in social anxiety disorder. Neuroimage. (2010) 52:1549–58. 10.1016/j.neuroimage.2010.05.010 20470894

[54] Kilts CD Kelsey JE Knight B Ely TD Bowman FD Gross RE. The neural correlates of social anxiety disorder and response to pharmacotherapy. Neuropsychopharmacology. (2006) 31:2243–53. 10.1038/sj.npp.1301053 16525417

[55] Watkins ER Roberts H. Reflecting on rumination: consequences, causes, mechanisms and treatment of rumination. Behav Res Therapy. (2020) 27:103573. 10.1016/j.brat.2020.103573 32087393

[56] Dey S Moorman J Moulds ML Newell BR. The relative effects of abstract versus concrete rumination on the experience of post-decisional regret. Behav Res Therapy. (2018) 108:18–28. 10.1016/j.brat.2018.06.007 29981935

[57] Hofmann SG. Cognitive factors that maintain social anxiety disorder: a comprehensive model and its treatment implications. Cogn Behav Ther. (2007) 36:193–209. 10.1080/16506070701421313 18049945 PMC2151931

[58] Nilsson JE Lundh LG Viborg G. Effects of analytical and experiential self-focus on rumination after a stress induction in patients with social anxiety disorder: a pilot study. Cog Behav Ther. (2012) 41:310–20. 10.1080/16506073.2012.682088 22536750

[59] Watkins E Moulds M. Distinct modes of ruminative self-focus: impact of abstract versus concrete rumination on problem solving in depression. Emotion. (2005) 5:319–28. 10.1037/1528-3542.5.3.319 16187867

[60] Dehaene S Naccache L. Towards a cognitive neuroscience of consciousness: basic evidence and a workspace framework. Cognition. (2001) 79:1–37. 10.1016/S0010-0277(00)00123-2 11164022

[61] Blackford JU Clauss JA Avery SN Cowan RL Benningfield MM VanDerKlok RM. Amygdala-cingulate intrinsic connectivity is associated with degree of social inhibition. Biol Psychol. (2014) 99:15–25. 10.1016/j.biopsycho.2014.02.003 24534162 PMC4274047

