# Supplementary material for: Alteration of resting-state functional connectivity network properties in patients with social anxiety disorder after virtual reality-based self-training
Source: Front Psychiatry. 2022 Sep 20;13:959696. doi: 10.3389/fpsyt.2022.959696 (PMC9530634; doi:10.3389/fpsyt.2022.959696)
Supplement: Supplementary file 1 [file Data_Sheet_1.DOCX]

Supplementary Material

# Additional details on image data preprocessing

## Anatomical data preprocessing

A total of 2 T1-weighted (T1w) images were found within the input BIDS dataset. All of them were corrected for intensity non-uniformity (INU) with N4BiasFieldCorrection (Tustison et al. 2010), distributed with ANTs 2.3.3 (Avants et al. 2008, RRID:SCR_004757). The T1w-reference was then skull-stripped with a Nipype implementation of the antsBrainExtraction.sh workflow (from ANTs), using OASIS30ANTs as target template. Brain tissue segmentation of cerebrospinal fluid (CSF), white-matter (WM) and gray-matter (GM) was performed on the brain-extracted T1w using fast (FSL 5.0.9, RRID:SCR_002823, Zhang, Brady, and Smith 2001). A T1w-reference map was computed after registration of 2 T1w images (after INU-correction) using mri_robust_template (FreeSurfer 6.0.1, Reuter, Rosas, and Fischl 2010). Volume-based spatial normalization to one standard space (MNI152NLin2009cAsym) was performed through nonlinear registration with antsRegistration (ANTs 2.3.3), using brain-extracted versions of both T1w reference and the T1w template. The following template was selected for spatial normalization: ICBM 152 Nonlinear Asymmetrical template version 2009c [Fonov et al. (2009), RRID:SCR_008796; TemplateFlow ID: MNI152NLin2009cAsym].

## Functional data preprocessing

For each of the 2 BOLD runs found per subject (across all tasks and sessions), the following preprocessing was performed. First, a reference volume and its skull-stripped version were generated using a custom methodology of fMRIPrep. Susceptibility distortion correction (SDC) was omitted. The BOLD reference was then co-registered to the T1w reference using flirt (FSL 5.0.9, Jenkinson and Smith 2001) with the boundary-based registration (Greve and Fischl 2009) cost-function. Co-registration was configured with nine degrees of freedom to account for distortions remaining in the BOLD reference. Head-motion parameters with respect to the BOLD reference (transformation matrices, and six corresponding rotation and translation parameters) are estimated before any spatiotemporal filtering using mcflirt (FSL 5.0.9, Jenkinson et al. 2002). BOLD runs were slice-time corrected using 3dTshift from AFNI 20160207 (Cox and Hyde 1997, RRID:SCR_005927). The BOLD time-series (including slice-timing correction when applied) were resampled onto their original, native space by applying the transforms to correct for head-motion. These resampled BOLD time-series will be referred to as preprocessed BOLD in original space, or just preprocessed BOLD. The BOLD time-series were resampled into standard space, generating a preprocessed BOLD run in MNI152NLin2009cAsym space. First, a reference volume and its skull-stripped version were generated using a custom methodology of fMRIPrep. Several confounding time-series were calculated based on the preprocessed BOLD: framewise displacement (FD), DVARS and three region-wise global signals. FD was computed using two formulations following Power (absolute sum of relative motions, Power et al. (2014)) and Jenkinson (relative root mean square displacement between affines, Jenkinson et al. (2002)). FD and DVARS are calculated for each functional run, both using their implementations in Nipype (following the definitions by Power et al. 2014). The three global signals are extracted within the CSF, the WM, and the whole-brain masks. Additionally, a set of physiological regressors were extracted to allow for component-based noise correction (CompCor, Behzadi et al. 2007). Principal components are estimated after high-pass filtering the preprocessed BOLD time-series (using a discrete cosine filter with 128s cut-off) for the two CompCor variants: temporal (tCompCor) and anatomical (aCompCor). tCompCor components are then calculated from the top 2% variable voxels within the brain mask. For aCompCor, three probabilistic masks (CSF, WM and combined CSF+WM) are generated in anatomical space. The implementation differs from that of Behzadi et al. in that instead of eroding the masks by 2 pixels on BOLD space, the aCompCor masks are subtracted a mask of pixels that likely contain a volume fraction of GM. This mask is obtained by thresholding the corresponding partial volume map at 0.05, and it ensures that components are not extracted from voxels containing a minimal fraction of GM. Finally, these masks are resampled into BOLD space and binarized by thresholding at 0.99 (as in the original implementation). Components are also calculated separately within the WM and CSF masks. For each CompCor decomposition, the k components with the largest singular values are retained, such that the retained components’ time series are sufficient to explain 50 percent of variance across the nuisance mask (CSF, WM, combined, or temporal). The remaining components are dropped from consideration. The head-motion estimates calculated in the correction step were also placed within the corresponding confounds file. The confound time series derived from head motion estimates and global signals were expanded with the inclusion of temporal derivatives and quadratic terms for each (Satterthwaite et al. 2013). Frames that exceeded a threshold of 0.5 mm FD or 1.5 standardised DVARS were annotated as motion outliers. All resamplings can be performed with a single interpolation step by composing all the pertinent transformations (i.e. head-motion transform matrices, susceptibility distortion correction when available, and co-registrations to anatomical and output spaces). Gridded (volumetric) resamplings were performed using antsApplyTransforms (ANTs), configured with Lanczos interpolation to minimize the smoothing effects of other kernels (Lanczos 1964). Non-gridded (surface) resamplings were performed using mri_vol2surf (FreeSurfer).

# Additional results on weighted network property analysis

In this section, we provide additional results on weighted network property analysis. The weighted networks are also computed from the MATLAB package gretna (Wang et al. 2015) with the *network type* option as *weighted.*

## Local network property analysis

Significant local network property analysis results for the weighted network are summarized in the Table S1. The findings demonstrate consistent result with the binarized network local properties, where the significant regions were revealed within the inferior frontal gyrus, dorsolateral prefrontal cortex (Brodmann area 9, 46), inferior temporal gyrus, Heschl’s gyrus, and calcarine sulcus.

## Global network property analysis

Significant global network property analysis result for the weighted network are summarized in the Table S2. The findings demonstrate consistent result with the binarized network global properties, where the significant interaction effect was found for the average shortest path length and the network efficiency, but without statistical significance in the post-hoc test.

**Table S1.** Significant results of the mixed-design ANOVA for the weighted local network metrics at different thresholds.

| **Region** | **Side** | **Network metrics** | **Threshold** | **F_1,37_** | ***p*-FDR** | **Post-hoc** | |
| --- | --- | --- | --- | --- | --- | --- | --- |
|  |  |  |  |  |  | **t_19_** | ***p*** |
| ***AAL*** | | | | | | | |
| Inferior frontal gyrus | Left | Nodal efficiency | 0.1 | 12.81 | 0.048 | 2.38 | 0.014 |
| Heschl's gyrus | Left | Clustering coefficient | 0.15 | 18.16 | 0.012 | 2.74 | 0.007 |
|  |  |  | AUC | 18.97 | 0.009 | 1.69 | 0.054 |
|  |  | Local efficiency | 0.15 | 17.62 | 0.015 | 2.62 | 0.008 |
|  |  |  | AUC | 17.45 | 0.016 | 1.60 | 0.063 |
|  |  | Nodal efficiency | 0.1 | 12.63 | 0.048 | 2.14 | 0.023 |
|  |  |  | AUC | 15.01 | 0.038 | 1.60 | 0.063 |
| Inferior temporal gyrus | Left | Degree centrality | 0.2 | 14.02 | 0.028 | 2.28 | 0.017 |
|  |  |  | 0.25 | 14.76 | 0.021 | 2.25 | 0.018 |
|  |  |  | 0.3 | 14.89 | 0.025 | 1.99 | 0.031 |
|  |  | Nodal efficiency | 0.2 | 14.81 | 0.041 | 1.74 | 0.049 |
| Calcarine sulcus | Right | Degree centrality | 0.2 | 17.22 | 0.017 | -2.53 | 0.010 |
|  |  |  | 0.25 | 18.37 | 0.011 | -2.54 | 0.010 |
|  |  |  | 0.3 | 14.34 | 0.025 | -2.31 | 0.016 |
| ***Glasser*** | | | | | | | |
| BA9/46d | Left | Degree Centrality | 0.35 | 16.95 | 0.037 | 3.23 | 0.002 |
|  |  |  | 0.4 | 17.53 | 0.032 | 3.24 | 0.002 |
|  |  |  | 0.45 | 18.02 | 0.044 | 3.25 | 0.002 |
|  |  |  | 0.5 | 18.45 | 0.044 | 3.24 | 0.002 |
|  |  | Nodal Efficiency | 0.2 | 18.76 | 0.039 | 3.53 | 0.001 |
| Ventromedial visual area 3 | Right | Clustering Coefficient | 0.3 | 18.11 | 0.049 | -2.30 | 0.016 |
| Anterior temporal area 2 | Right | Degree Centrality | 0.3 | 19.30 | 0.033 | -3.92 | <0.001 |
|  |  |  | 0.35 | 19.06 | 0.035 | -3.85 | 0.001 |
|  |  |  | 0.4 | 17.40 | 0.032 | -3.69 | 0.001 |
|  |  |  | 0.45 | 16.46 | 0.044 | -3.58 | 0.001 |
|  |  |  | AUC | 19.88 | 0.027 | -4.14 | <0.001 |
| BA9m | Left | Local Efficiency | 0.1 | 18.22 | 0.047 | 5.15 | <0.001 |

Abbreviation: AUC, Area under the curve; AAL, Automated Anatomical Labeling; BA9/46d, Dorsal part of the Brodmann area 9/46; BA9m, Middle part of the Brodmann area 9.

**Table S2.** Significant results of the mixed-design ANOVA for the weighted global network metrics at different thresholds.

| **Network metrics** | **Threshold** | **F_1,37_** | ***p*** | **Post-hoc** | |
| --- | --- | --- | --- | --- | --- |
|  |  |  |  | **t_19_** | ***p*** |
| ***AAL*** | | | | | |
| Network efficiency | 0.05 | 4.48 | 0.041 | 1.10 | 0.143 |
| Small-worldness | 0.05 | 4.86 | 0.034 | 0.77 | 0.226 |
| ***Glasser*** | | | | | |
| Network efficiency | 0.05 | 7.02 | 0.012 | 1.64 | 0.059 |
|  | 0.1 | 5.96 | 0.020 | 1.56 | 0.068 |
|  | 0.15 | 4.65 | 0.038 | 1.31 | 0.103 |
| Average shortest path length | 0.05 | 5.55 | 0.024 | -1.46 | 0.081 |
|  | 0.1 | 6.19 | 0.017 | -1.49 | 0.076 |
|  | 0.15 | 4.96 | 0.032 | -1.26 | 0.112 |

**References**

Abraham, Alexandre, Fabian Pedregosa, Michael Eickenberg, Philippe Gervais, Andreas Mueller, Jean Kossaifi, Alexandre Gramfort, Bertrand Thirion, and Gael Varoquaux. 2014. “Machine Learning for Neuroimaging with Scikit-Learn.” Frontiers in Neuroinformatics 8. https://doi.org/10.3389/fninf.2014.00014.

Avants, B.B., C.L. Epstein, M. Grossman, and J.C. Gee. 2008. “Symmetric Diffeomorphic Image Registration with Cross-Correlation: Evaluating Automated Labeling of Elderly and Neurodegenerative Brain.” Medical Image Analysis 12 (1): 26–41. https://doi.org/10.1016/j.media.2007.06.004.

Behzadi, Yashar, Khaled Restom, Joy Liau, and Thomas T. Liu. 2007. “A Component Based Noise Correction Method (CompCor) for BOLD and Perfusion Based fMRI.” NeuroImage 37 (1): 90–101. https://doi.org/10.1016/j.neuroimage.2007.04.042.

Cox, Robert W., and James S. Hyde. 1997. “Software Tools for Analysis and Visualization of fMRI Data.” NMR in Biomedicine 10 (4-5): 171–78. https://doi.org/10.1002/(SICI)1099-1492(199706/08)10:4/5<171::AID-NBM453>3.0.CO;2-L.

Esteban, Oscar, Ross Blair, Christopher J. Markiewicz, Shoshana L. Berleant, Craig Moodie, Feilong Ma, Ayse Ilkay Isik, et al. 2018. “FMRIPrep.” Software. Zenodo. https://doi.org/10.5281/zenodo.852659.

Esteban, Oscar, Christopher Markiewicz, Ross W Blair, Craig Moodie, Ayse Ilkay Isik, Asier Erramuzpe Aliaga, James Kent, et al. 2018. “fMRIPrep: A Robust Preprocessing Pipeline for Functional MRI.” Nature Methods. https://doi.org/10.1038/s41592-018-0235-4.

Fonov, VS, AC Evans, RC McKinstry, CR Almli, and DL Collins. 2009. “Unbiased Nonlinear Average Age-Appropriate Brain Templates from Birth to Adulthood.” NeuroImage 47, Supplement 1: S102. https://doi.org/10.1016/S1053-8119(09)70884-5.

Gorgolewski, K., C. D. Burns, C. Madison, D. Clark, Y. O. Halchenko, M. L. Waskom, and S. Ghosh. 2011. “Nipype: A Flexible, Lightweight and Extensible Neuroimaging Data Processing Framework in Python.” Frontiers in Neuroinformatics 5: 13. https://doi.org/10.3389/fninf.2011.00013.

Gorgolewski, Krzysztof J., Oscar Esteban, Christopher J. Markiewicz, Erik Ziegler, David Gage Ellis, Michael Philipp Notter, Dorota Jarecka, et al. 2018. “Nipype.” Software. Zenodo. https://doi.org/10.5281/zenodo.596855.

Greve, Douglas N, and Bruce Fischl. 2009. “Accurate and Robust Brain Image Alignment Using Boundary-Based Registration.” NeuroImage 48 (1): 63–72. https://doi.org/10.1016/j.neuroimage.2009.06.060.

Jenkinson, Mark, Peter Bannister, Michael Brady, and Stephen Smith. 2002. “Improved Optimization for the Robust and Accurate Linear Registration and Motion Correction of Brain Images.” NeuroImage 17 (2): 825–41. https://doi.org/10.1006/nimg.2002.1132.

Jenkinson, Mark, and Stephen Smith. 2001. “A Global Optimisation Method for Robust Affine Registration of Brain Images.” Medical Image Analysis 5 (2): 143–56. https://doi.org/10.1016/S1361-8415(01)00036-6.

Lanczos, C. 1964. “Evaluation of Noisy Data.” Journal of the Society for Industrial and Applied Mathematics Series B Numerical Analysis 1 (1): 76–85. https://doi.org/10.1137/0701007.

Power, Jonathan D., Anish Mitra, Timothy O. Laumann, Abraham Z. Snyder, Bradley L. Schlaggar, and Steven E. Petersen. 2014. “Methods to Detect, Characterize, and Remove Motion Artifact in Resting State fMRI.” NeuroImage 84 (Supplement C): 320–41. https://doi.org/10.1016/j.neuroimage.2013.08.048.

Reuter, Martin, Herminia Diana Rosas, and Bruce Fischl. 2010. “Highly Accurate Inverse Consistent Registration: A Robust Approach.” NeuroImage 53 (4): 1181–96. https://doi.org/10.1016/j.neuroimage.2010.07.020.

Satterthwaite, Theodore D., Mark A. Elliott, Raphael T. Gerraty, Kosha Ruparel, James Loughead, Monica E. Calkins, Simon B. Eickhoff, et al. 2013. “An improved framework for confound regression and filtering for control of motion artifact in the preprocessing of resting-state functional connectivity data.” NeuroImage 64 (1): 240–56. https://doi.org/10.1016/j.neuroimage.2012.08.052.

Tustison, N. J., B. B. Avants, P. A. Cook, Y. Zheng, A. Egan, P. A. Yushkevich, and J. C. Gee. 2010. “N4ITK: Improved N3 Bias Correction.” IEEE Transactions on Medical Imaging 29 (6): 1310–20. https://doi.org/10.1109/TMI.2010.2046908.

Wang J, Wang X, Xia M, Liao X, Evans A, He Y. “GRETNA: a graph theoretical network analysis toolbox for imaging connectomics.” Frontiers in Human Neuroscience (2015) 9:386.

Zhang, Y., M. Brady, and S. Smith. 2001. “Segmentation of Brain MR Images Through a Hidden Markov Random Field Model and the Expectation-Maximization Algorithm.” IEEE Transactions on Medical Imaging 20 (1): 45–57. https://doi.org/10.1109/42.906424.
